# Millisecond, Micron Precision Multi-Whisker Detector

**DOI:** 10.1371/journal.pone.0073357

**Published:** 2013-09-02

**Authors:** Stephen K. Grady, Thanh T. Hoang, Shree Hari Gautam, Woodrow L. Shew

**Affiliations:** Department of Physics, University of Arkansas, Fayetteville, Arkansas, United States of America; Instituto de Neurociencias de Alicante UMH-CSIC, Spain

## Abstract

The neural mechanisms of somatosensory information processing in the rodent vibrissae system are a topic of intense debate and research. Certain hypotheses emphasize the importance of stick-slip whisker motion, high-frequency resonant vibrations, and/or the ability to decode complex textures. Other hypotheses focus on the importance of integrating information from multiple whiskers. Tests of the former require measurements of whisker motion that achieve high spatiotemporal accuracy without altering the mechanical properties of whiskers. Tests of the latter require the ability to monitor the motion of multiple whiskers simultaneously. Here we present a device that achieves both these requirements for two-dimensional whisker motion in the plane perpendicular to the whiskers. Moreover, the system we present is significantly less expensive (<$2.5 k) and simpler to build than alternative devices which achieve similar detection capabilities. Our system is based on two laser diodes and two linear cameras. It attains millisecond temporal precision and micron spatial resolution. We developed automated algorithms for processing the data collected by our device and benchmarked their performance against manual detection by human visual inspection. By this measure, our detection was successful with less than 10 µm deviation between the automated and manual detection, on average. Here, we demonstrate its utility in anesthetized rats by measuring the motion of multiple whiskers in response to an air puff.

## Introduction

Whiskers provide essential sensory information for many mammals. The computational strategies of the vibrissae system are among the most vigorously researched topics in sensory neuroscience. Numerous experimental strategies have been developed for precise measurement and control of whisker motion. The most common approach is to attach the whisker to a device, the motion of which is precisely controlled [1]–[12]. However, this approach raises concerns that the attached device alters the mechanical properties of the whisker. Since mechanical properties, such as inertia and resonant frequencies [13], [14], whisker flexibility and curvature [15], may play a role in somatosensation, it is desirable to develop whisker motion detectors which do not mechanically contact the whisker. Moreover, it is difficult to be certain that an attached device does not maintain an ongoing and unwanted force on the whisker due to holding the whisker slightly away from its natural resting position. This approach is also not useful for studies of active whisking in awake animals.

An alternative approach is to monitor the position of the whisker optically. To this end, some employ high-speed video cameras [13]–[21], which have the benefit of providing information on whisker curvature as well as position. Another key advantage of camera based systems is that they can provide a view of multiple whiskers simultaneously, which is necessary for understanding how potentially different information from different vibrissae is integrated [1]–[3], [21]. With few exceptions [14], most camera-based systems employ a single camera, thus capturing two dimensions of whisker motion. Since cameras are most often positioned above the animal, looking down, motion along the medial-lateral direction is often not captured. This is unsuitable for some studies of whisker-direction-selectivity of neurons [2]. Roy et al. (2011) overcame this problem using a multi-camera motion capture system to measure three dimensional whisker motion, but their system required that a reflective bead to be attached to the whisker which may alter the mechanical properties of the whisker [22]. Moreover, the motion capture system was somewhat limited in time resolution (200 frames/sec). Although this may be sufficiently temporally precise for the large-scale motion of active whisking in air, contact with the environment will induce whisker motion that occurs on faster time scales. For example, as a rodent sweeps its whisker across a textured surface, the resulting motion is a stick-slip trajectory with slip events on 100 µm and ms timescales [13], [23]. Moreover, such contact with the environment will induce vibrations of the whisker with resonant frequencies up to 250 Hz [13], [14].

An alternative to cameras is to use simpler optical sensors to capture the shadow cast by the whisker, which is positioned between the sensors and a light source. The system we report here adopts this approach. Before we describe our system, we review features of other previously reported similar devices. Using a single optical sensor, Lottem and Azouz (2008, 2009) were able to capture very small scale vibrations (micron scale) of single whiskers with high accuracy near the whisker follicle, but this approach is not suitable for studying large scale motions (e.g. natural whisking) or multiple whiskers [24], [25]. Other groups have employed a linear array of optical sensors which enables a larger range of observed motions. Variations on this approach have been implemented by several groups with success [13], [26], [27]. Spatial resolution on the scale of a few microns and temporal resolution around 1 ms have been achieved. With two such systems, one can easily capture two dimensions of whisker motion. Importantly, the two dimensions that are typically (and most easily) captured are in a plane perpendicular to the whisker, i.e. both the rostral-caudal and medial-lateral directions, which may be important for studying whisker-direction selective neurons. Whisker motion in the third dimension, away-towards the mystacial pad, is less likely to occur. An important advantage of this approach is that it is significantly less expensive than previously reported high-speed cameras or multi-camera motion-capture systems. Disadvantages include the fact that whisker curvature is not obtained and that these systems often require non-trivial in-house design and assembly of optics related to the light source and electronics to read from the linear light sensor. The light sensors used by Wolfe et al. (2008), to our knowledge, are no longer commercially available. Moreover, the system used by Bermejo et al. (1998, 2002), requires that a small object be attached to the whisker to facilitate detection, which may be problematic as mentioned above. Perhaps the most important disadvantage of previously reported systems which use linear optical sensors is that the detection electronics were designed to identify single whiskers only. This precludes the study of hypotheses related to the integration of information from multiple whiskers [1]–[3], [21].

Here we describe a system which is also based on a pair of linear light sensors, but is capable of monitoring the motion of multiple whiskers with millisecond temporal resolution, micron spatial resolution, and does not require any objects to be attached to the whiskers. It is very simple to construct, with minimal expertise in optics or electronics required. Moreover, the system we describe is very low cost - $2.5 k, which is less than half the cost of previously reported similar systems and many thousands of dollars less expensive than camera based systems. Our system has the potential to illuminate how the sensory information gained from different whiskers, potentially including whisker-specific resonant vibrations, is integrated to form a holistic somatosensory ‘view’ of the environment.

## Methods

### Ethics Statement

All procedures were carried out in accordance with the recommendations in the Guide for the Care and Use of Laboratory Animals of the National Institutes of Health. The protocol was approved by University of Arkansas Institutional Animal Care and Use Committee (protocol #12025).

The whisker detector we describe in the next section was demonstrated with measurements of neural activity in barrel cortex in response to whisker stimulation of adult male rats (300–400 g; *Rattus Norvegicus*, Sprague-Dawley outbred, Harlan Laboratories, TX, USA). Rats were initially anesthetized with isoflurane inhalation. Anesthesia was maintained with an intraperitoneal (ip) injection of urethane (1.5 g/kg body weight (bw) dissolved in saline). Surgery was begun after a subcutaneous injection of lidocaine (2%, 0.2 ml) in the scalp and injections of dexamethasone (2 mg/kg bw, ip) and atropine sulphate (0.4 mg/kg bw, ip). The scalp was cut and the skull was cleaned before attaching a head-bar with dental cement. A craniotomy was performed over barrel cortex and the dura was cut. The craniotomy was rectangular spanning 1 to 3 mm posterior from bregma and 5 to 7 mm lateral from midline.

The rat was positioned with its whiskers (contralateral to craniotomy) within the detector described below and the head bar was fixed to a rigid support. All except 1 to 4 whiskers were trimmed. A Michigan-style electrode array (4 shanks with 8 electrodes each, 200 µm inter-electrode distance, 400 µm inter-shank distance, A4×8-5 mm-200–400-177-A32, NeuroNexus, MI, USA) was inserted into barrel cortex using a 1-axis electro-mechanical micro-manipulator (Burleigh 6000 ULN, Thorlabs Inc, NJ, USA) attached to a manual micro-manipulator (1760–61, David Kopf Instruments, CA, USA). Electrodes were inserted such that the electrode plane was perpendicular to the pial surface and parallel to the midline. The array spanned 1.6 mm along the rostro-caudal direction and was inserted to a depth of 650–700 µm. The center of the electrode array was 2.5 mm posterior from bregma and 6 mm lateral from midline. After insertion, small gel foam pieces soaked in artificial cerebral spinal fluid were placed around the electrodes to keep the brain wet. An AgCl pellet electrode was placed in the gel foams to serve as ground and reference.

The recorded extracellular voltages were digitized with the Cereplex digital head stage and recorded with the Cerebus system (Blackrock Microsystems, UT, USA). This system also recorded the scan signals of the two whisker sensors and a trigger signal for the air puff used to stimulate the whiskers, as described further below.

## Device and Results

**Figure 1:**
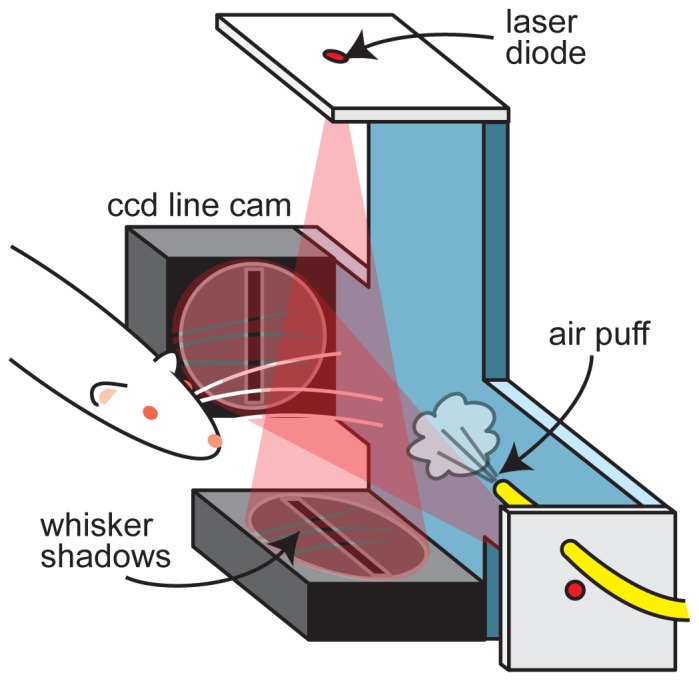
Device schematic. Rostro-caudal and medial-lateral motion of one or more whiskers is detected based on shadows cast by the whiskers upon two CCD line cameras (up to 1000 images/sec, 1×2048 pixels, 14 µm/pixel resolution). The light sources are laser diodes. An air puff was used to stimulate whisker motion.

The whisker detector is based on two linear charge-coupled devices (CCD) each with 2048 pixels in a line and the capability to acquire one linear ‘image’ per 1.06 milliseconds (LC100, Smart Line Camera, Thorlabs Inc, NJ, USA). These devices interface with a data acquisition computer via USB and are supplied with Labview drivers, allowing easy integration into a complex experimental system. We include Labview code for controlling the CCDs in Supporting Information (S1). The CCDs are configurable to provide a 0–5 V square pulse for each image acquired, thus facilitating precise temporal alignment with other data acquisition systems used to record, for example, neural data. Each pixel spans 14 microns, providing excellent spatial resolution over the 2.87 cm length of each CCD. As shown in Figure 1, a laser diode (650 nm, 5 mW, D650-5I, US Lasers Inc, CA, USA) positioned 17 cm from each CCD provides coherent point-source illumination of the entire CCD. Two CCDs and two laser diodes are mounted to a rigid frame as shown in Figure 1.

For the purposes of demonstrating the utility of this system, we used pressure-controlled air puffs to stimulate the whiskers. It would not be difficult to implement other types of stimulation, e.g. a rotating, or translating textured surface. It is also plausible to integrate this device into a behavioral experiment in which active whisking is studied as in previous studies where the rat is trained to whisk in a specific location (e.g. [13]). Our puffer system included a computer-controlled pressure regulator (IP610-030, Omega Engineering Inc., CT, USA) connected to a compressed air source. Following the pressure regulator was a small electro-mechanical valve (LHDA0531115H, The Lee Company, CT, USA). The pressure regulator, the valve, and data acquisition from the CCDs were controlled with a Labview program and a NI 6343 acquisition system (National Instruments, TX, USA). The puff pressure was tunable from zero to 30 PSI and the valve could be opened for any duration, allowing a wide range of puff intensities. The examples shown in this paper were 0.5 sec in duration with a puff tube inner diameter of 1.5 mm positioned 11 cm from the whisker. Repeated delivery of the same puff intensity never results in precisely the same whisker motion due to unpredictable turbulent motions of the puffed air. Other stimulation means may also create some degree of trial-to-trial variability in induced whisker motion. This is why it is crucial to have a system, like the one we describe here, for precise measurement of whisker motion for every stimulation trial.

Laser diodes were chosen for a light source for two reasons: 1) they provide coherent point-source illumination and 2) they were very inexpensive (<$20). Coherent point-source illumination is useful for two reasons. First, the divergence of the light cone amplifies the motion of the whisker, increasing spatial resolution. Second, coherent light is diffracted by the whisker creating a predictable diffraction pattern shadow on the CCD. This diffraction pattern serves two important purposes. First, it facilitates the detection of thin whiskers (e.g. mouse whiskers), which have been difficult to detect with incoherent light [22]. Second, as described in more detail below, the distinct wavy diffraction pattern allows the correct detection of whisker positions even when the whisker shadows spatially overlap on the CCD.

**Figure 2:**
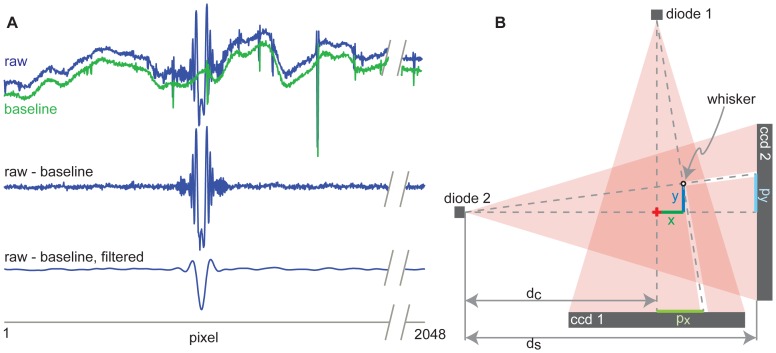
Obtaining whisker position from line camera data. **A)** A single scan from one line camera (top). The spatial irregularities of the raw scan are due primarily to non-uniform illumination from the diodes. These irregularities are removed by subtracting a baseline scan (green), which is obtained with no whisker present. After subtracting the baseline scan, the whisker shadow waveform is clearly visible (middle). Finally, the scan is low-pass filtered so that the minimum point precisely identifies the pixel at the center of the whisker shadow (bottom). **B)** The pixel positions of the shadows (*p_x_* and *p_y_*) are then used to compute the actual spatial positions (x and y): *x* = (*d_c_* - *p_y_ d_c_*/*d_s_*)/(*d_s_*/*p_x_*+*p_y_*/*d_s_*) and *y* = *p_y_* (*d_c_*+*x*)/*d_s_*. These formulas are geometrically derived from the diagram shown here.

**Figure 3:**
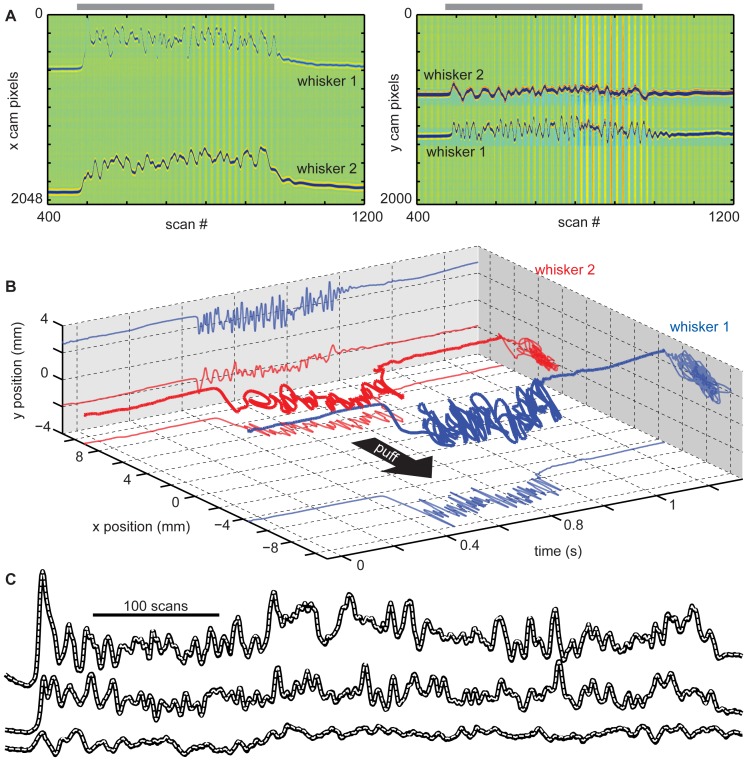
Multiple whisker detection. **A)** Line camera data versus time for two whiskers during one air puff. The horizontal axis (scan #) represents time (1 scan per 2 ms). The vertical axis represents pixel (x line cam – left; y line cam – right). Color represents light intensity (arb. units, after subtracting reference and filtering, see Fig 2). Gray bars indicate duration of puff. **B)** The x and y positions of whisker 1 (blue) and whisker 2 (red) versus time. The heavy line shows the 2D trajectory. Thin lines are projections (x(t) – bottom; y(t) – left; x(y) – right) of the trajectory. The puff direction is indicated with the black arrow. **C)** Accuracy of automatically detected positions (black) for 3 whiskers is confirmed by comparing with manually detected positions (dashed, white). Miniscule differences (0.9±0.8 pixels) between the automatic and manual detections demonstrate the reliability of the automatic detection algorithm.

Even without a whisker, the laser diodes do not produce spatially uniform illumination of the CCDs. (There were no significant changes in intensity over time.) Thus the first step of data analysis is to subtract this baseline illumination pattern from the image, as demonstrated in Figure 2A. The next step is a low-pass filter to remove higher spatial frequencies and reveal a clear minimum intensity, which identifies the pixel at the center of the whisker shadow. These steps are carried out with the images from both CCDs. Finally, to obtain the actual whisker position, some simple geometry is applied as illustrated in Figure 2B. To obtain whisker position with good temporal resolution during the entire puff stimulation, the CCDs are sampled at up to 993 images/sec. Each image is treated as described in Figure 2 to obtain the whisker trajectories as demonstrated in Figure 3. Matlab code for fast multi-whisker detection from a sequence of many scans (<1 s for analyzing 1000 scans) is included in Supporting Information (S2). As is apparent in Figure 3, the motions of the puffed whisker can be complex, highlighting the importance of monitoring both the rostral-caudal and the medial-lateral axes of motion. Figure 3 also demonstrates the ability of our system to monitor multiple whiskers simultaneously. We have successfully tracked up to 4 whiskers with this device and data analysis.

To quantify the success of our automatic detection algorithm, we compared to manual detection based on visual inspection. Manual detection was done by two people separately (not including the person who wrote the algorithm). A separate Matlab program was used to display the light intensity profile for each scan and the human detector was instructed to click on center of each whisker shadow. This was done for 1000 scans during which 3 whiskers were in motion due to an air puff. The automatic detection was finished in less than 1 s, while the manual detection required at least 3 hrs of clicking. The automatically detected whisker positions never deviated by more than 8 pixels (approximately 100 µm) from the manual detections and 99% of automatic detections were within 3 pixels of the manual detections. The average difference between the automatic and manual detections was 0.9±0.8 (mean±SD) pixels. For comparison, the two humans differed from each other by 1.3±1.0 pixels with 99% of differences within 4.6 pixels. Thus we conclude that the automatic detection algorithm is highly reliable.

When multiple whiskers are studied, an important step in the experiment is to match up each whisker with its corresponding shadow positions on both line cameras. For the anesthetized animal experiments we have carried out here, it is easy to do this step manually – e.g. displace one whisker from the imaged volume and note which shadow disappears from each CCD. This approach would also work for awake, head-fixed animals (e.g. [15]). If the device was used for freely moving animals, single whiskers could easily be studied (assuming the animal was trained to whisk within the detector), but multiple whisker studies would require careful trimming the whiskers such that there is no ambiguity about which whiskers create which shadows.

**Figure 4:**
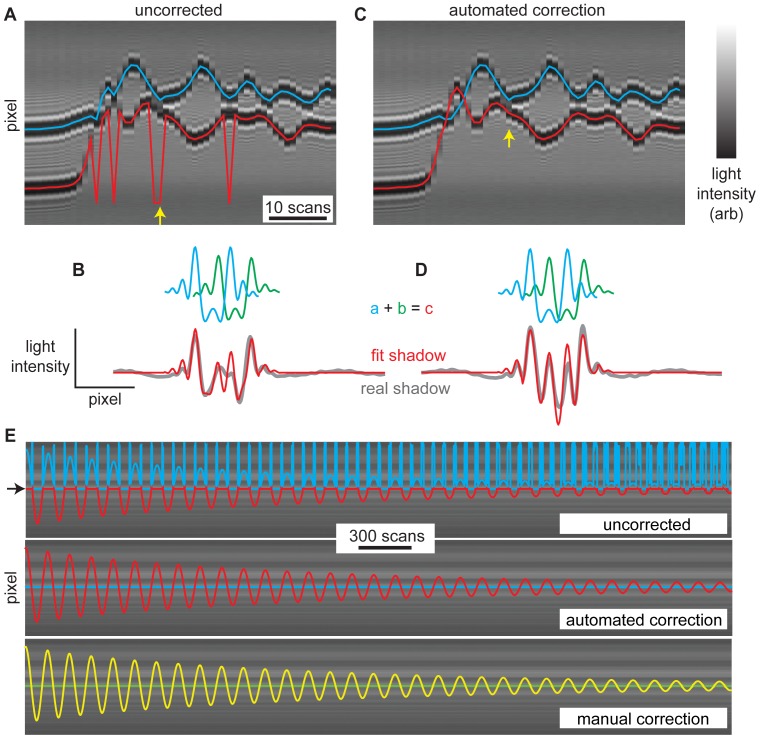
Distinguishing whiskers with overlapping shadows. **A)** Data from one line camera versus time for two whiskers during motion which results in overlapping whisker shadows. Whisker motion was caused by an air puff. Detecting whisker positions based on shadow minima fails when shadows overlap. The red and blue lines show incorrectly detected positions of the two whiskers. The horizontal axis represents time (1 scan per 2 ms). The vertical axis represents pixel. Grayscale represents light intensity. **B)** The overlapping shadow positions are correctly detected by fitting a linear combination (red) of non-overlapping shadow waveforms (blue, green) to the real shadow (gray). The yellow arrow in A indicates the scan which is corrected here. **C)** The same data shown in A after correcting all the scans in which shadows overlapped using the technique illustrated in B. **D)** Another example of using a linear combination of shadow waveforms to detect positions of overlapping shadows. This scan is not shown in A and C. **E)** A second example of data from one line camera versus time for two whiskers during motion which results in overlapping whisker shadows. One whisker was stationary at the pixel marked with an arrow. The other whisker was attached to a mechanical oscillator. Whisker trajectories are shown before correction (top) and after automated correction (middle), and after manual correction by a human (bottom). The difference between the automated and manual correction was 1.0±1.3 pixels (mean±SD), demonstrating agreement with approximately 10 µm precision.

An additional challenge can arise when studying multiple whiskers if motion results in shadows which spatially overlap each other on the CCD. Non-overlapping whisker shadows are necessary to use the Matlab code provided in Supporting Information S2. The air puff that we use tends to cause all the whiskers to deflect in largely the same direction. By choosing well separated whiskers, this typically results in whisker shadows which do not spatially overlap each other. However, since other types of whisker stimulation may generate whisker motion that results in overlapping shadows, we have developed an alternative data analysis approach which works well in this case. Matlab code for this alternative analysis is included in Supporting Information (S3). We considered two test cases to demonstrate this approach. First, we did an experiment with two whiskers which were positioned such that our air puff resulted in overlapping shadows. Second, we created a dataset in which one whisker was stationary while a second whisker was attached to an oscillating device. Here both whiskers were detached from the rat and attached to mechanical devices. For these cases the approach described in Figure 2 fails to correctly identify whisker positions because the two whisker shadows overlap in such a way that together then generate only one minimum, as shown in Figure 4A for the puffed whiskers. Our alternative approach takes advantage of the fact that the laser diode light sources create reliable complex shadow waveforms due to diffraction of light by the whiskers. First, we run the algorithm (S2) for non-overlapping whiskers. Then, the algorithm (S3) identifies the first scan in which detection failed. Failed detections are automatically determined based on an ongoing prediction of whisker positions based on past whisker position and velocity. More specifically, the predicted position *p(t)* at time *t* is equal to the position at time *t-1* plus a small change Δ*p*, *p(t)* = *p(t-1)+*Δ*p*, where Δ*p* is based on the average whisker speed over the previous 3 time steps Δ*p = (p(t-1) – p(t-4))/3dt.*

Any scan which deviates from the prediction by more than a threshold (e.g. 60 pixels) is considered a ‘failed detection’ and then is corrected as follows. The first step of the correction analysis is to obtain a pure shadow waveform for each whisker when there are no overlapping shadows. This is only done once per data set. Next, we identify the linear combination of the pure waveforms which best fits the real shadow (Figure 4B). Before determining the best fit, the pure waveforms are corrected based on whisker velocity before fitting. This correction is essential when the whiskers move at high speeds, because, in this case, the pure waveforms become blurred due to the distance moved during one scan exposure. For low whisker speeds, this step is not necessary. For *n* whiskers, there are *n* fitting parameters – one for the spatial position of each pure shadow waveform. In principle, all possible positions of all whiskers could be included in the search for best fit, but the algorithm is much faster if we make the following two improvements: 1) we restrict our search to positions within 70 pixels of the position in the previous scan, and 2) first do a coarse search (5 pixel resolution) followed by a fine search (single pixel resolution) near the best fit from the course search. After a scan is corrected using this fitting procedure, the algorithm moves on to the next failed detection. This process is repeated until there are no more failed detections.

This method worked well for both our test cases as shown in Figure 4. As with the non-overlapping whisker detection algorithm, we quantified the success of our algorithm compared to manual detection based on visual inspection. We carried out this comparison for the oscillating whiskers test case, which was comprised of 4000 scans. Manual detection was done by two people separately (not including the person who wrote the algorithm). Manual detection required 15–20 hrs. The results of one person are shown in Figure 4E. Like the algorithm, the people were allowed to view the recent history of the whisker motion to aid in their manual detection. The results of the algorithm were extremely similar to the manually detected trajectories. The difference between automated and manually detected trajectories was 1.0±1.3 pixel, which is not quite as small as for the easier, non-overlapping case, but still very small. For comparison, the difference between the manually detected trajectories of the two people was 0.7±1.1 pixels. A difference of 1 pixel corresponds to less than 14 µm and the thickness of a rat whisker is 50–100 µm.

Compared to the detection algorithm for non-overlapping whiskers (S2), the algorithm for overlapping whiskers (S3) is much slower and may require more tuning of parameters.

The algorithm for overlapping whiskers required approximately 1 min, which is approximately 10–20 times slower than the non-overlapping algorithm. Thus, depending on the number of scans in which the whisker shadows overlap, this technique can be very time consuming. Moreover, the non-overlapping algorithm requires no adjustment of parameters by the user, while the overlapping algorithm may benefit from tuning the threshold for determining when a scan is not properly detected and the resolution of the coarse fitting step.

**Figure 5:**
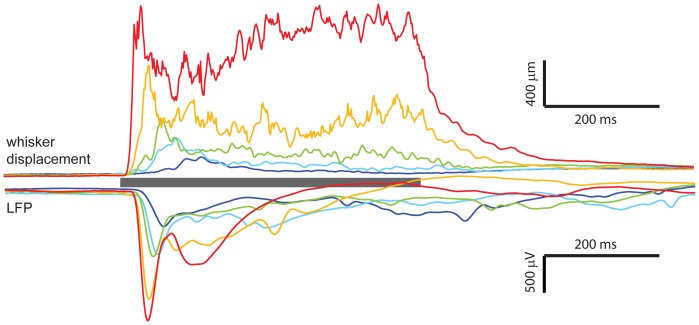
Neural response to complex whisker motion. **(top)** Average whisker deflection for 5 different puff strengths (from weak to strong: dark blue, cyan, green, yellow, red). Average is over 10 repeats for each puff strength. Gray bar indicates time of puff. **(bottom)** Average extracellular field potential (0–500 Hz) recorded during the corresponding whisker motion shown above. Average is over 10 repeats for each puff strength and 32 recording locations spanning superficial layers of barrel cortex with bicuculline methiodide applied locally at recording site (20 µM in saline at pia).

Importantly, this system provides plenty of space around the rat for electrophysiology instrumentation. To demonstrate this, we present extracellular field potential recordings (filtered 0–500 Hz) performed simultaneously with the whisker motion detection (Figure 5) in a urethane-anesthetized adult male rat (Methods). The recordings in Fig 5, are averaged over 32 electrodes inserted in superficial layers. GABA_A_ antagonist bicuculline methiodide (Sigma-Aldrich 14343) was applied to the surface of the cortex where the electrodes were implanted. Such treatment is known to expand the receptive fields and increase response to whisker stimulation among neurons recorded in barrel cortex [28]. The bicuculline methiodide was dissolved in saline to a concentration of 20 µM. Gel foam pieces were soaked in this solution and placed in contact with the brain surrounding the electrode insertion site.

## Discussion

In conclusion, we describe a device for detecting the position of many whiskers simultaneously with millisecond, micron precision. We have successfully used this device to measure the motion of up to 4 whiskers in response to a puff of air while recording electrophysiological signals from barrel cortex. In our experience, the device and the analysis (S2) we present work without fail provided a few conditions are met. First, the whisker motion must not produce shadows which spatially overlap. If the shadows overlap, it is still feasible to analyze the data and obtain whisker positions (Figure 4), but this analysis is time consuming and relatively difficult. We note that in our studies with air puff whisker stimulation, it was easy to trim most whiskers leaving intact 3 to 4 whiskers which do not have overlapping shadows. A simpler practical concern is that care must be taken to keep the scanners clean and maintain fixed ambient lighting conditions. If hair or debris falls on the scanner during an experiment spurious shadows can preclude easy whisker detection analysis. If ambient lighting changes and the baseline scan (Figure 2) does not account for these changes, whisker detection is more difficult. Finally, we note that we have had success using this device to detect whiskers from rows A-C and arcs 1–4. We have not attempted to study whiskers α, β, γ, δ, because they protrude in a primarily caudal direction, nor rows D and E, nor arcs 5–7 because these whiskers tend to be short and more difficult to position in the scanned volume.

Advantages of our device include its low cost compared to alternative high-speed videography techniques and its simplicity to construct and use. Another important feature of our system is that the whiskers are free to move without any objects attached, which may be necessary for studies of how mechanical properties of whiskers play a role in somatosensory coding. We anticipate that this system will be useful for the study of how different sensory information obtained by different whiskers is integrated to encode complex textures and dynamics of a rodent’s environment.

## Supporting Information

Figure S1 **Labview code for controlling linear CCD cameras.** With a camera plugged into a USB port, this code will acquire images from the camera.
(VI) Click here for additional data file.

Figure S2 **Matlab code for basic multiple whisker detection.** This code is fast and works very well in the case where the whisker shadows never overlap or cross each other.
(M) Click here for additional data file.

Figure S3 **Matlab code for difficult multiple whisker detection.** This code is slower. It handles the case where the whisker shadows overlap and cross each other.
(M) Click here for additional data file.
